# The Impact of Proactive Fecal Calprotectin Collection in an Outreach Protocol for Biologic-Naïve Ulcerative Colitis Patients–Ulcerative Colitis Clinical Outreach (UCCO)

**DOI:** 10.3390/diseases14010002

**Published:** 2025-12-22

**Authors:** Scott MacKay, Denise Parsons, Candace Hagerman, Ellina Lytvyak, Levinus Dieleman, Frank Hoentjen, Karen Kroeker, Farhad Peerani, Karen Wong, Michal Gozdzik, Kunihiko Oguro, Todd McMullen, Brendan Halloran

**Affiliations:** 1 Division of Gastroenterology, University of Alberta, Edmonton, AB T6G 2X8, Canada; samacka1@ualberta.ca (S.M.); deparson@ualberta.ca (D.P.); levinus@ualberta.ca (L.D.); hoentjen@ualberta.ca (F.H.); karen.kroeker@ualberta.ca (K.K.); kwong3@ualberta.ca (K.W.); gozdzik@ualberta.ca (M.G.); oguro@ualberta.ca (K.O.); 2 Division of Emergency Medicine, University of Alberta, Edmonton, AB T6G 2X8, Canada; candace.hagerman@albertahealthservices.ca; 3 Division of Preventive Medicine, University of Alberta, Edmonton, AB T6G 2X8, Canada; lytvyak@ualberta.ca; 4 Department of Medicine, Division of Gastroenterology, Jichi Medical University, Shimotsuke 329-0498, Tochigi, Japan; 5 Division of General Surgery, University of Alberta, Edmonton, AB T6G 2X8, Canada; tpm1@ualberta.ca

**Keywords:** ulcerative colitis, remote outreach, fecal calprotectin

## Abstract

Background: Ulcerative colitis (UC) is a chronic, relapsing inflammatory bowel disease that requires regular monitoring. The University of Alberta IBD Unit piloted a proactive outreach protocol for biologic-naïve UC patients, including clinical and biochemical variables, and assessed its impact on UC care. Methods: Biologic-naïve UC patients without follow-up for ≥6 months were recruited by phone and completed Partial Mayo, modified Sutherland Index, and MARS-5 questionnaires, as well as blood work and fecal calprotectin (FCP). Results were sent to each patient’s gastroenterologist, who then completed a survey about intended UC management changes. Results: 81 patients completed the protocol. UC management was changed in 45 (55.6%) cases, with 82.2% of changes being expedited follow-up or management escalation. Six patients had active flares, and 17 with asymptomatic inflammation were identified. 23 patients underwent endoscopy, with 10 (43.4%) showing active disease. Six patients started biologic therapies based on protocol and endoscopic findings. UC management escalations were significantly predicted by FCP and Sutherland Index scores on logistic regression analysis. 86.4% of gastroenterologists rated the protocol helpful. Conclusions: Patient care can be improved by a one-time, proactive outreach program for biologic-naïve UC. Outreach and monitoring in biologic-naïve UC should include assessment of both FCP and clinical markers to improve UC management.

## 1. Introduction

Ulcerative colitis (UC) is a chronic, relapsing inflammatory bowel disease (IBD) localized to the colon [1]. UC has estimated annual costs of 8.1–14.9 billion dollars in the United States and 5.38 billion dollars in Canada [2,3]. For mild to moderate UC, there is often less urgency to achieve and confirm mucosal healing, and patients do not require appointments to receive biologic therapies, leaving them prone to limited adherence [4,5,6]. UC can place a large symptom burden on patients, ranging from intestinal symptoms (i.e., bloody diarrhea, tenesmus, and abdominal pain) to various extraintestinal manifestations and sequelae of systemic inflammation [1]. These symptoms typically correspond with colonic inflammation, though inflammation can persist in asymptomatic patients and is associated with worse disease outcomes regardless of baseline disease severity [4]. When patients are not experiencing symptoms they deem burdensome, they may decrease adherence to medications, avoid or postpone appointments with their gastroenterologist, or be lost to follow-up [4]. This can undermine effective management of UC, which requires regular monitoring and long-term maintenance therapy to maintain disease remission and in turn reduce the personal and economic burdens of flares, colectomies, colorectal cancer, and overall morbidity of UC [1,4,5,7,8]. Furthermore, resource limitations in many practices cause follow-up to be delayed or sparse if patients are asymptomatic and considered stable.

UC disease severity is assessed clinically, biochemically, and via the gold standard method of direct tissue visualization with endoscopy and colon biopsies [8]. Many clinical disease activity indices for UC exist, though the two most frequently used remain the Mayo Clinic score and Sutherland Index [1,9]. Both indices calculate scores based on stool frequency, presence of rectal bleeding, appearance of colonic mucosa on endoscopy, and a physician assessment of disease severity to stratify UC as mild, moderate, severe, or in remission. Biochemical markers influenced by UC include C-reactive protein (CRP), erythrocyte sedimentation rate (ESR), ferritin, platelets, leukocytes, albumin, and procalcitonin as acute phase reactants, which can become abnormal in the setting of systemic inflammation, as well as fecal calprotectin (FCP) as a more specific marker of gastrointestinal inflammation [8]. Endoscopic and histologic appearances also have multiple scoring indices used to stratify disease severity, with the Mayo Endoscopic Score remaining the most commonly used index for endoscopy [8,10].

Telemedicine and mobile health (mHealth) programs in IBD management have demonstrated cost-effectiveness and improved clinical outcomes for UC patients [11,12,13,14,15]. Monitoring programs studied to date have generally focused on clinical indices and selected for patients already closely followed in IBD clinics. One study explored rates of healthcare utilization related to monitoring in UC, though the specific clinical decision-making that is made by IBD physicians in response to monitoring programs has not been explored to date [11]. The International Organization for the Study of IBD’s STRIDE-II recommendations include frequent monitoring of clinical indices and biomarkers (i.e., C-reactive protein (CRP) and fecal calprotectin (FCP)) concurrently to guide management changes and determine if patients have reached defined therapeutic targets [16]. Simulated models have demonstrated higher rates of disease remission, reduced flares, and lower economic burden for mild to moderate UC when monitoring protocols include both clinical indices and biomarkers [5,17].

In 2018, our center developed and piloted a proactive clinical outreach protocol for adult, biologic-naïve UC patients to determine disease activity with clinical indices and biomarkers and assess the resulting UC management decisions made by their IBD physicians. We sought patients who had not been in contact with our center or their gastroenterologist in at least six months to assess the impact of the protocol in patients both at risk of being lost to follow-up and perceived as being stable. Further, we specifically sought biologic-naïve patients, as this population is commonly considered low risk and, contrary to those on biologic therapies, does not require monitoring to maintain medication coverage nor appointments for medication infusions where concerns can be raised. The aim of our study was to assess the effectiveness of our proactive outreach protocol for guiding UC management.

## 2. Materials and Methods

### 2.1. Study Design

Our study was a single-center, nonrandomized interventional study with our monitoring protocol as the intervention and IBD physician decision-making as the outcome. The study ran from November 2016 to July 2018 with continuous recruitment throughout from the outpatient practices of six IBD physicians.

### 2.2. Patients

Electronic health records were reviewed to identify biologic-naïve adult UC patients with no history of colectomy who had not contacted our center or their gastroenterologist in at least six months. Patients similarly had not presented to the hospital with concerns of UC flares or active UC. All patients were followed by the University of Alberta IBD Unit located in Edmonton, AB, Canada.

### 2.3. Protocol

Eligible patients were mailed an FCP test kit and blood work requisition, with specimens sent to a provincial laboratory for testing. Questionnaires assessing clinical indices and medication compliance were also provided for participants to complete. Subjective clinical activity was assessed using a partial Mayo score and a modified Sutherland index, both of which omitted scoring related to endoscopic findings [17,18]. A 5-item Medication Adherence Report Scale (MARS-5) was completed to assess adherence to medications prescribed for UC with a cut-off score of ≥21 to determine adherence [19,20]. Requested blood work included complete blood count, CRP, iron, ferritin, creatinine, albumin, alkaline phosphatase, and alanine transaminase. FCP was collected to assess gastrointestinal inflammation.

On the basis of clinical indices scores and FCP levels, patients were categorized into four subgroups: (1) active flares (i.e., at least moderate symptoms and FCP ≥ 250 µg/g), (2) symptomatic (i.e., at least mild symptoms and not meeting criteria for flare), (3) asymptomatic inflammation (i.e., clinical remission and FCP ≥ 250 µg/g), and (4) remission (clinical remission and FCP < 150 µg/g). A threshold of FCP < 150 µg/g was chosen for the remission group, as this cut-off was previously identified as an indicator of endoscopic healing [14].

The results of these questionnaires and lab tests were compiled in a letter, which was sent to the patient’s gastroenterologist for review. After providing informed consent, gastroenterologists then completed a survey asking if the letter was helpful and to identify any UC management changes they planned to make in response to the letter. All IBD specialists at our center participated in the study. Management changes were categorized as UC follow-up expeditions and management escalations (i.e., booking clinic appointments, booking endoscopy appointments, ordering non-endoscopic investigations, changing medication class or dose) or follow-up delays (i.e., delaying or canceling upcoming appointments). A second retrospective chart review was conducted from August 2023 to October 2023 to assess whether proposed management changes were completed and the results of investigations, as well as treatment escalations made in response to endoscopy.

### 2.4. Outcomes

The primary outcomes of this study were to (1) assess the disease status of presumed stable, biologic-naïve UC outpatients with clinical indices and biomarkers and (2) assess if the protocol resulted in changes to UC management. A secondary objective was to assess which variables predicted UC changes to inform future monitoring protocols in UC management.

### 2.5. Statistical Analysis

Descriptive data was analyzed using means and standard deviations. Shapiro–Wilk testing was completed for all numerical variables to confirm normality. Associations between variables were assessed using Pearson correlation analysis, and comparisons between groups were completed using independent-samples T-tests and one-way ANOVA analyses. Logistic regression analysis was performed to determine which variables predicted escalation of the UC management. For all tests, significance was assessed at *p* < 0.05. Analysis was conducted using IBM SPSS Statistics for Windows, version 29.0 (IBM Corp., Armonk, NY, USA).

## 3. Results

### 3.1. Baseline Patient Characteristics

A total of 101 patients were contacted, with 18 (17.8%) providing no answer and 2 (2.0%) declining to participate. 81 adult UC patients (63.0% female) with a mean (SD) age of 52.4 (13.67) years (range 23–81 years) completed the protocol. Patient characteristics are summarized in Table 1.

### 3.2. Medication Adherence

Using the MARS-5 questionnaire, 57 patients (86.4%) were found to be adherent to medications and the remaining 9 (13.6%) showed decreased adherence. Non-adherent patients were significantly younger than adherent patients (mean age: 42.0 vs. 54.0 years, *p* = 0.006).

### 3.3. Disease Activity

#### 3.3.1. Clinical Indices

Twenty-seven patients (33.3%) reported at least mild clinical disease activity. Table 1 summarizes the proportion of patients in each grade of clinical disease severity. Partial Mayo and Sutherland Index scores were positively correlated with each other (*r* = 0.60, *p* < 0.01) and concordant in terms of symptom severity in 70.4% of cases.

#### 3.3.2. Hematological and Biochemical Parameters

Blood work results are summarized in Table 2. The decision to expedite follow-up or escalate UC management was significantly correlated with CRP (*r* = 0.26, *p* < 0.01), MCV (*r* = −0.28, *p* < 0.05), and serum iron (*r* = −0.24, *p* < 0.05) levels. There were no significant correlations between CRP and FCP, nor CRP and active disease on endoscopy.

#### 3.3.3. FCP

The median value for FCP in our protocol patients was 142.0 µg/g with an overall mean (SD) value of 379.18 µg/g (658.10 µg/g). Twenty-three patients (28.4%) had FCP levels ≥ 250 µg/g. FCP levels was positively correlated with Partial Mayo (*r* = 0.35, *p* < 0.01), modified Sutherland Index (*r* = 0.32, *p* < 0.01), and endoscopic Mayo (*r* = 0.73, *p* < 0.01) scores. FCP levels at each level of disease severity on these scales were significantly different when compared in a one-way ANOVA analysis (Table 3).

### 3.4. Physician Surveys

Six IBD physicians from the University of Alberta IBD Unit took part in this study. The protocol letter was rated as helpful for UC management for 70 of these patients (86.4%).

#### Management Changes

**Figure 1:**
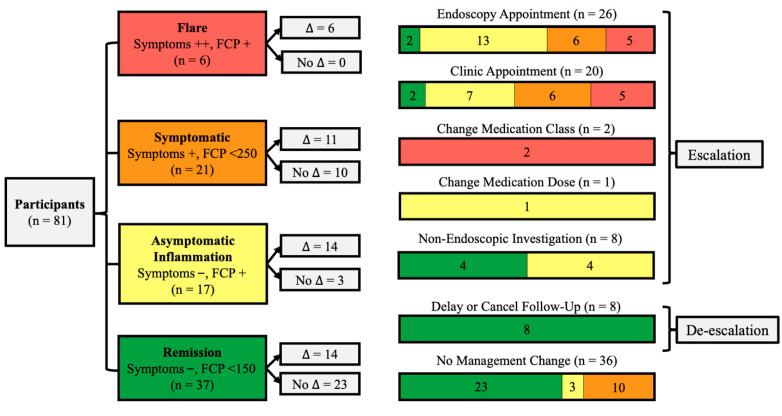
Ulcerative Colitis Management Changes by Clinically Relevant Patient Subgroups. Legend. FCP = Fecal calprotectin. Δ = Management change made. Symptoms: − indicates no symptoms, + indicates at least mild disease (i.e., Partial Mayo (≥2) or Modified Sutherland Index (≥2)), ++ indicates at least moderate disease (i.e., Partial Mayo (≥5) or Modified Sutherland Index (≥6)). FCP: + indicates level ≥ 250 μg/g, all values listed in units of μg/g.

UC management was changed for 45 patients (55.6%) in response to the protocol. Figure 1 summarizes the changes made for the overall sample and each clinically relevant subgroup. The mean time from IBD physician survey completion to booked clinic appointment was 7.31 months. Of the 8 patients with further investigations ordered, four (50%) had repeat blood work ordered, 3 (37.5%) had “other” tests (i.e., FCP and azathioprine metabolites) ordered, and 1 (12.5%) had diagnostic imaging (i.e., CT Enterography) ordered. All patients who had their medication class changed were started on infliximab, and the patients who had their medication dose changed had their 5-ASA dose increased. The mean delay of follow-up was 8.57 months.

In total, 37 (82.2%) of the 45 patients whose UC management was changed had follow-up expedited or management escalated. These patients had significantly greater mean partial Mayo scores (1.41 vs. 0.39, *p* = 0.007), modified Sutherland index scores (3.49 vs. 1.68, *p* < 0.001), FCP levels (713.0 vs. 123.4 µg/g, *p* < 0.001), and CRP levels (6.96 vs. 3.04 mg/L, *p* = 0.024) than those with unchanged UC management or delayed follow-up. Conversely, these patients had significantly lower mean levels of serum iron (13.95 vs. 17.24 umol/L, *p* = 0.031), ferritin (75.19 vs. 112.92, *p* = 0.045), and MCV (87.86 vs. 91.11, *p* = 0.015).

### 3.5. Endoscopy

Endoscopy was booked for 26 patients and completed in 23 patients. The remaining three patients declined the procedure. Table 1 summarizes the proportion of patients in each grade of endoscopic disease severity using the Mayo Endoscopic score [10]. In all 23 cases, an infectious stool work-up was sent either prior to or at the time of endoscopy and returned negative. Endoscopic findings led to 4 further biologic therapy starts (2 vedolizumab, 1 infliximab, 1 adalimumab), 3 rectal topical therapy starts, 3 budesonide tapers, and 4 changes to 5-ASA therapies (1 start, 1 re-initiation of therapy, 1 dose increase, and 1 switch in 5-ASA formulation). Ten patients had no management changes made after the endoscopy was completed.

### 3.6. Regression

Logistic regression was completed to assess for variables that predicted escalations in UC management. This analysis demonstrated that FCP levels (Exp(*β*) = 1.003, *p* = 0.023) and Sutherland Index scores (Exp(*β*) = 3.203, *p* = 0.044) were the only significant predictors of UC management escalation. This model predicted correctly in 75.0% of cases and had a Nagelkerke R-squared value of 0.440, meaning approximately 44% of the variance in UC management escalation decisions was predicted by the model. Two logistic regression models were subsequently run examining the same variables with FCP dichotomized to cut-offs of 150 µg/g and 250 µg/g. In both cases, FCP and Sutherland Index scores remained significant predictors of UC management escalation. Table 4 summarizes all three logistic regression models.

### 3.7. Clinically Relevant Subgroups

Six patients (7.1%) were found to be in active disease flares. These patients had a mean (SD) FCP value of 1401.0 µg/g (664.2 µg/g) and a median FCP of 1247.0 µg/g. All six had their UC management escalated, including both patients who started biologic therapies. Five (83.3%) completed endoscopy with a range of mild (n = 2), moderate (n = 2), and severe (n = 1) disease seen. Endoscopic findings led to additional management escalations, with two biologic starts (one on adalimumab, one on vedolizumab), one budesonide taper, and one 5-ASA dose escalation. The remaining patient had Mayo 1 disease on endoscopy and had no management changes made in response to these findings.

A group of 17 patients with asymptomatic inflammation defined by FCP levels (≥250 µg/g) and clinical remission on partial Mayo (n = 16) and/or modified Sutherland Index (n = 11) scores was also identified. Mean (SD) FCP levels of 797.6 µg/g (599.2 µg/g) and 734.91 µg/g (660.2 µg/g) were found for those in remission on partial Mayo and Sutherland Index scores, respectively. Median FCP values for these groups were 533.0 µg/g and 360.0 µg/g, respectively. UC management escalations and expedited follow-ups were ordered for 14 of these patients (82.4%). One patient had their oral 5-ASA dose increased, and thirteen were booked for endoscopy. Mild (n = 4), moderate (n = 3), and severe (n =1) disease was seen on endoscopy, leading to one biologic start (vedolizumab), one budesonide start, and one addition of oral 5-ASA to a previously rectal-only regimen. The remaining five patients had no management changes made after endoscopy.

## 4. Discussion

We report on outcomes of our proactive clinical outreach assessment for biologic-naïve UC patients. Despite the presumption that participating patients were stable in the outpatient setting, we identified 27 patients with at least mild clinical disease, 17 patients with asymptomatic inflammation, and a subgroup of 6 patients with active flares. These findings led to management changes in 55.6% of UC patients, and the protocol was reported as helpful by IBD physicians in 82.4% of cases. These changes were mostly expedited follow-ups or management escalations, driven by evidence of active disease on modified Sutherland Index scores and elevated FCP levels. As a result of this protocol and subsequent endoscopy, six patients were escalated to biologic therapies. These findings demonstrate the important role proactive monitoring can play in detecting active inflammation in mild-to-moderate UC, allowing for earlier intervention. The extent of disease and opportunities for UC management optimization we identified at a single time point suggests that regular, proactive monitoring of UC biomarkers and clinical scores is important for biologic-naïve UC patients.

Over 40 percent of patients were in remission clinically and biochemically. These findings were similarly useful for IBD physicians, as they allowed for delaying or canceling follow-up appointments. The ability of an outpatient monitoring program to guide these decisions can reduce scheduling burdens on IBD clinics and the transportation and lost productivity costs for patients [13]. This also allows for patients with clinically active disease to be seen in these clinic spots, potentially resulting in more timely assessment and management.

From a UC outreach perspective, a particularly relevant subset of patients identified in our study was the 17 patients with asymptomatic inflammation. UC has typically been thought to be a disease of less asymptomatic inflammation, and outpatient monitoring studies to date have almost exclusively focused on clinical indices [15]. The absence of biomarkers in UC monitoring leaves patients with asymptomatic inflammation susceptible to being undertreated and creates uncertainty about the achievement of therapeutic targets outlined by STRIDE-II recommendations [16]. To our knowledge, only a 2019 study from McCombie et al. utilized both clinical activity indices and biomarker (i.e., FCP) measurements in an outpatient monitoring study for IBD patients, and their sample contained predominantly Crohn’s disease patients [14]. Two recent studies have further demonstrated the effectiveness of monitoring both clinical symptoms and FCP in patients with mild to moderate UC by utilizing computer-simulated models [5,17]. Our study demonstrated that a combination of clinical indices and biomarkers directly influenced management decisions by IBD physicians for real-world patients with mild to moderate UC.

Active disease on Sutherland Index scores and FCP levels significantly predicted UC management escalation on logistic regression analysis. Sutherland Index scores were significantly correlated with Partial Mayo scores, and both scores have well-established clinical utility [9,10]. CRP, MCV, ferritin, and serum iron levels were significantly different between patients who had UC management escalations made and those who did not. Despite this, FCP was identified as the only significant predictor of UC management escalation in logistic regression analysis, which is in keeping with a growing area of UC literature examining the clinical value of FCP and its potential for use as a singular biomarker of disease activity in UC monitoring programs [21,22,23,24,25].

The use of FCP has become commonplace in UC care, and as outpatient UC monitoring continues to advance, home collection kits and smartphone apps have been developed for remote FCP monitoring [26,27,28,29]. These apps utilize smartphone cameras to quantify calprotectin in stool samples using a rapid immunochromatographic assay approach and have been shown to have high patient usability, with good correlation and clinical application comparable to traditional ELISA laboratory assays [28]. These home collection methods are most accurate at FCP levels < 500 µg/g, which is particularly relevant for more subtle inflammation in patients that may be in clinical remission [29,30]. The ability to remotely monitor FCP accurately and reliably is valuable for UC monitoring programs, particularly for centers with large rural and remote patient populations.

Our study is limited in generalizability as our participant population was exclusively patients with biologic-naïve UC from a single academic IBD center and the clinical decision-making and perceived helpfulness of the monitoring protocol came from a sample of six IBD physicians. The single-time-point disease assessment is a limitation, as patient adherence to monitoring over time could not be assessed. However, this also demonstrates that even a single outreach assessment can be helpful in UC patients who are otherwise assumed to be doing well by their treating physicians. The absence of a control group in this study does limit potential statistical analyses, though a control group was not feasible as our participants were not actively in contact with their IBD physician, and therefore no clinical decision-making was being made for them before our intervention. Our study used directed phone calls for both patient recruitment and assistance with completing the provided protocol as opposed to a completely remote platform. Requiring personnel to complete phone calls could be a barrier for implementation in some IBD clinics and could reduce economic benefits compared to a fully remote protocol. That said, having a clear point of contact likely had the benefit of ensuring questionnaire completion and increasing patient compliance, without which, monitoring programs are of no value.

Future research exploring outpatient IBD outreach programs could benefit from further exploration of telemedicine and mHealth approaches, particularly with concurrent at-home FCP collection. A cost-effectiveness analysis and economic modeling of this approach would also be meaningful when developing a basis for larger-scale implementation. Similarly, a longitudinal or prospective study examining an IBD monitoring program to compare outcomes with standard of care monitoring and assess patient adherence would be important. Ongoing research into the accuracy and reliability of home FCP collections will also benefit this area given the importance of biomarkers in UC patients in clinical remission.

## 5. Conclusions

This outreach protocol had a meaningful impact on biologic-naïve UC patients who were presumed stable in the outpatient setting, directly impacting management changes by IBD physicians in 55.6% of cases and identifying patients with active disease in both the presence and absence of clinical remission. Our findings highlight the importance of collecting both FCP and clinical disease scores proactively to detect asymptomatic inflammation in mild to moderate UC. Our analysis demonstrated that FCP was the only biomarker that predicted UC management escalations, suggesting that outpatient monitoring could potentially focus on this single measure to limit the cost of a monitoring program and the testing burden on patients. Outreach and remote monitoring protocols provide an opportunity for comprehensive care from IBD centers to reach patients regularly via telemedicine visits and potentially improve their disease outcomes by detecting and acting on active disease in a preventative manner.

## Figures and Tables

**Table 1:** Participant Characteristics.

Age (Years)	Mean		SD		Range
	52.4		13.67		23–81
**Sex**		**Frequency**		**Percentage (%)**	
Female		51		63.0	
Male		30		37.0	
**Medications** ^a^					
Oral 5-ASA ^b^		68		84.0	
Azathioprine		10		12.3	
Topical Rectal Therapies ^c^		15		18.5	
FNo Medications		15		18.5	
**Last Follow-up**					
6 Months to 1 Year		43		53.1	
1 Year to 2 Years		22		27.2	
>2 Years		16		19.8	
**Partial Mayo Score (*n* = 81)**					
Remission (<2)		64		79.0	
Mild (2–4)		13		16.0	
Moderate (5–6)		2		2.5	
Severe (7–9)		2		2.5	
**Endoscopic Mayo Score (*n* = 22)**					
No Active Disease (0)		4		18.2	
Mild (1)		9		40.9	
Moderate (2)		6		27.2	
Severe (3)		3		13.6	
**Modified Sutherland Index (*n* = 81)**					
Remission (0–2)		54		66.7	
Mild (3–5)		17		21.0	
Moderate (6–11)		10		12.3	
Severe (12+)		0		0	
**MARS-5 Questionnaire (*n* = 66)**					
Adherent (>20)		57		86.4	
Non-adherent (=< 20)		9		13.6	

^a^ Listed medications are not mutually exclusive for patients. ^b^ Oral 5-ASA includes Asacol, Salofalk, Pentasa, and Mezavant. ^c^ Includes 5-ASA and corticosteroid rectal therapies. MARS-5 = Medication Adherence Report Scale.

**Table 2:** Baseline Laboratory Results.

Laboratory Results (n = 81)	Normal Values ^a^	Median	Mean	SD
Fecal Calprotectin	<50 µg/g	142.00	379.18	658.10
C-Reactive Protein	<8.0 mg/L	1.75	4.85	7.41
Hemoglobin *	120–160 g/L	142.00	140.44	11.93
Mean Corpuscular Volume	80–100 fL	90.00	89.80	6.08
Leukocytes	4.0–11.0 × 10^9^/L	6.40	6.89	2.03
Platelets	140–400 × 10^9^/L	252.00	254.86	68.59
Serum Iron *	8–35 umol/L	14.00	15.69	6.74
Ferritin *	20–300 umol/L	68.00	93.98	81.52
Creatinine *	40–100 umol/L	75.00	76.69	20.01
Albumin	30–45 g/L	43.00	42.84	2.56
Alkaline Phosphatase	40–120 U/L	73.00	86.57	46.76
Alanine Transaminase	<50 U/L	26.00	31.54	25.87

^a^ Values listed are the normal values reported by the laboratories that analyzed each test. For hemoglobin, ferritin, creatinine, and alanine transaminase these ranges are for female patients. * Variables with a non-normal distribution on Shapiro–Wilk testing.

**Table 3:** Comparing Fecal Calprotectin by Ulcerative Colitis Disease Severity.

**Partial Mayo** **Category**	**Remission** **(n = 64)**		**Mild** **(n = 13)**		**Moderate** **(n = 2)**		**Severe** **(n = 2)**		**F** **(4, 81)**	**η2**
	**Median**	**Mean (SD)**	**Median**	**Mean (SD)**	**Median**	**Mean (SD)**	**Median**	**Mean (SD)**		
FCP	109.0	257.0 (419.2)	267.0	753.7 (1149.8)	1247.0	1247.0 (169.7)	1231.5	1231.5 (1706.2)	5.12 *	0.16
**Sutherland Index** **Category**	**Remission** **(n = 54)**		**Mild** **(n = 17)**		**Moderate** **(n = 10)**		**Severe**		**F** **(2, 81)**	**η2**
	**Median**	**Mean (SD)**	**Median**	**Mean (SD)**	**Median**	**Mean (SD)**	**Median**	**Mean (SD)**		
FCP	72.5	217.4 (403.5)	197.0	530.5 (888.2)	782.5	872.1 (843.7)	n/a	n/a	5.52 *	0.12
**Endoscopic Mayo** **Category**	**No Active Disease** **(n = 4)**		**Mild** **(n = 9)**		**Moderate** **(n = 7)**		**Severe** **(n = 3)**		**F** **(2, 16)**	**η2**
	**Median**	**Mean (SD)**	**Median**	**Mean (SD)**	**Median**	**Mean (SD)**	**Median**	**Mean (SD)**		
FCP	243.0	415.3 (356.7)	361.0	497.1 (364.2)	804.0	1034.9 (766.9)	2438.0	2821.7 (1201.4)	13.02 *	0.65

* Significant at *p* < 0.01. FCP = fecal calprotectin (µg/g), SD = standard deviation. Partial Mayo Scores: 0–1 (Remission), 2–4 (Mild), 5–6 (Moderate), 7–9 (Severe). Sutherland Index Scores: 0–1 (Remission), 2–5 (Mild), 6–11 (Moderate), 12+ (Severe). n/a is stated for the Severe category of the Sutherland Index as no participants were found to have severe disease using this questionnaire.

**Table 4:** Logistic Regression of Factors Predicting Ulcerative Colitis Management Escalation.

Variable	*β*	S.E.	Exp(*β*)	95% CI	*p*	*R* ^2^ *_N_*
FCP	0.003	0.001	1.003	1.000, 1.006	0.023	
CRP	0.058	0.042	1.060	0.977, 1.149	0.163	
Sutherland Index Active Disease	1.164	0.579	3.203	1.030, 9.959	0.044	
						0.440
FCP (≥250 µg/g)	2.482	0.647	11.964	3.300, 43.382	<0.001	
CRP	0.070	0.043	1.072	0.986, 1.166	0.104	
Sutherland Index Active Disease	1.396	0.583	4.039	1.288, 12.660	0.017	
						0.473
FCP (≥150 µg/g)	1.763	0.556	5.830	1.962, 17.326	0.002	
CRP	0.056	0.038	1.057	0.980, 1.140	0.148	
Sutherland Index Active Disease	1.288	0.557	5.357	1.218, 10.798	0.021	
						0.394

*R*^2^*_N_* = Nagelkerke R Square. Sutherland Index Active Disease is a dichotomous variable categorized as remission or active disease of any severity. FCP (≥250 µg/g) is a dichotomous variable categorized as FCP ≥ 250 µg/g or <250 µg/g. FCP (≥150 µg/g) is a dichotomous variable categorized as FCP ≥ 150 µg/g or <150 µg/g.

## Data Availability

The data underlying this article cannot be shared publicly due for the privacy of individuals that participated in the study. The data will be shared on reasonable request to the corresponding author.

## Abbreviations

The following abbreviations are used in this manuscript:

CRP	C-reactive protein
FCP	Fecal calprotectin
IBD	Inflammatory Bowel Disease
mHealth	Mobile health
UC	Ulcerative Colitis
